# From vascular normalization to VEGF-independent escape: multi-omics-defined angio-immune ecosystem states

**DOI:** 10.3389/fcell.2026.1788177

**Published:** 2026-07-03

**Authors:** Baolai Li, Haitao Song, Yongmei Dai, Xiaofeng Wu

**Affiliations:** Department of Obstetrics, Qingdao Hospital, University of Health and Rehabilitation Sciences (Qingdao Municipal Hospital), Qingdao, Shandong, China

**Keywords:** Immune exclusion, myeloid immunosuppression, Vascular normalization, Vasculogenic mimicry, Vessel co-option

## Abstract

Anti-angiogenic therapy and immune checkpoint blockade can synergize, yet benefits are frequently transient, implying that resistance reflects ecosystem-level reprogramming rather than failure of a single pathway. Here we define angio-immune ecosystem states as reproducible tumor microenvironment configurations that couple vascular function, immune composition, and spatial architecture. We summarize four resistance-associated states: endothelial–stromal gatekeeping with immune exclusion, a time-limited normalization window, hypoxia/myeloid-driven rebound immunosuppression, and VEGF-independent perfusion escape via vessel co-option, vasculogenic mimicry, or intussusceptive remodeling. Finally, we propose an interpretable measurement stack, integrating bulk transcriptional modules, single-cell–anchored cell-state programs, and spatial neighborhood features, to enable cross-cohort stratification and longitudinal tracking. This framework supports state-matched combinations by linking therapeutic resistance to distinct spatial configurations of the intravascular lumen, endothelial interface, perivascular niche, stromal–parenchymal boundary, and immune-cell trafficking axis.

## 1 Introduction

The tumor microenvironment (TME) is increasingly viewed as a dynamic ecosystem in which vascular organization and immune contexture co-evolve, shaping progression and therapeutic vulnerability (Lanitis et al., 2025; Liu et al., 2019). Beyond tumor-intrinsic genomics, clinical outcomes (particularly for immunotherapy) are often determined by how this ecosystem is functionally and spatially structured around malignant cells (Huang et al., 2012; Fukumura et al., 2018). In this setting, the vasculature is not merely a passive conduit for oxygen and drug delivery; it actively organizes immune trafficking, endothelial “gatekeeping,” perivascular niche formation, stromal remodeling, and metabolic constraints such as hypoxia and acidosis, all of which can condition antigen presentation, T-cell positioning, and effector function (Pitt et al., 2016). Accordingly, resistance to immune checkpoint blockade (ICB) and to anti-angiogenic therapy frequently reflects emergent ecosystem adaptations rather than a single, targetable pathway failure (Wang X. et al., 2025).

Anti-angiogenic agents were initially designed to starve tumors by disrupting neovascularization, yet vascular remodeling can exert bidirectional effects on antitumor immunity. In favorable contexts, partial vascular normalization improves perfusion and oxygenation, reduces extreme leakiness, and facilitates immune entry—creating a transient therapeutic window in which ICB and other immune modalities can be more effective (Jain, 2001; Xu and Shao, 2024). However, immune entry after vascular modulation should not be interpreted as a simple linear consequence of improved perfusion. Whether normalization translates into effective antitumor immunity depends on spatially coordinated events, including leukocyte adhesion within the vascular lumen, endothelial chemokine and adhesion-molecule presentation, perivascular immune-cell retention or release, and the ability of T cells to cross the stromal–parenchymal boundary and reach malignant-cell neighborhoods (Khan and Kerbel, 2018). This window is often short-lived and strongly context dependent, varying with dose, schedule, tumor type, and the baseline vascular–immune landscape (Shen et al., 2022). This context dependence is especially relevant in gynecologic oncology (e.g., cervical, ovarian, and endometrial cancers), where angiogenic stress and hypoxia commonly coexist with stromal/vascular gatekeeping that limits effective immune entry. Outside the optimal range, excessive pruning can intensify hypoxia and stress signaling, reinforcing immunosuppressive programs and spatial immune exclusion. Thus, the same class of vascular intervention may either enable or obstruct immune-mediated control depending on how the ecosystem evolves over time.

A major obstacle to durable benefit from anti-angiogenic–immunotherapy combinations is the emergence of adaptive resistance configurations. These include endothelial–stromal barrier states that restrict adhesion and transendothelial migration (Lanitis et al., 2015; Ribatti, 2024a); myeloid-dominated rebound programs in which macrophages and other suppressive myeloid populations restore pro-angiogenic cues while dampening cytotoxic immunity (Dal et al., 2017; Wang M. et al., 2025); and VEGF-independent perfusion strategies that bypass canonical sprouting angiogenesis. In particular, vessel co-option and vasculogenic mimicry provide clinically relevant escape routes, maintaining blood supply by hijacking pre-existing host vessels or forming perfusable tumor-cell–lined channels, respectively (Seliger and Massa, 2021). Such adaptations are often accompanied by architectural shifts that are poorly captured by bulk averages alone.

This mini-review advances a spatially organized, state-centric explanation for transient and heterogeneous responses to vascular–immune combinations. Rather than treating angiogenesis, hypoxia, and immune exclusion as separable processes, we define angio-immune ecosystem states as integrated tissue configurations that couple vascular function, endothelial gatekeeping, perivascular niche composition, stromal–parenchymal barriers, and immune-cell trafficking (Ribatti, 2024b). In this framework, the key question is not simply whether anti-angiogenic therapy increases immune infiltration, but under which spatial and molecular conditions vascular remodeling converts immune exclusion into productive intratumoral immunity, and when it instead promotes hypoxia-driven rebound or VEGF-independent escape. We therefore organize the review around four resistance-associated ecosystem states and discuss how bulk, single-cell, and spatial readouts can be used to define, track, and therapeutically intercept these transitions. The novelty of this review is not the general notion that the TME functions as an ecosystem, but the explicit organization of vascular–immune resistance into four spatially defined, therapeutically interpretable ecosystem states. By integrating the normalization-window concept with spatial immune positioning, endothelial-state heterogeneity, VEGF-independent vascular escape, and a practical bulk–single-cell–spatial measurement stack, this framework aims to convert descriptive vascular–immune biology into state-matched therapeutic reasoning.

## 2 Conceptual framework: spatially organized angio-immune ecosystem states

**FIGURE 1:**
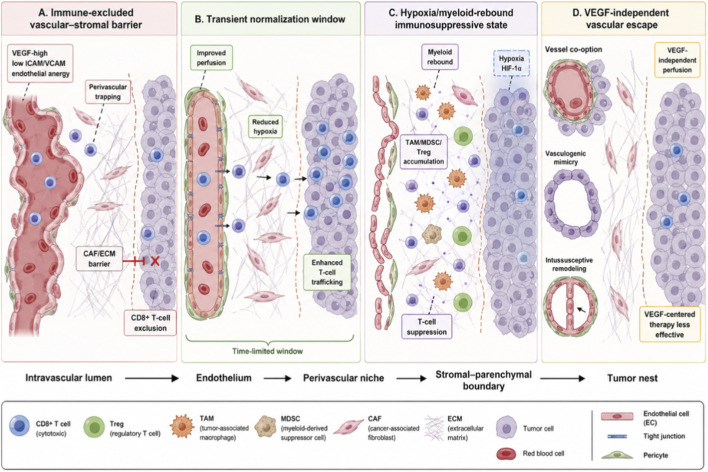
Spatial organization of vascular–immune ecosystem states. **(A)** Immune-excluded vascular–stromal barrier state: abnormal vessels, endothelial anergy, and CAF/ECM barriers restrict CD8^+^ T-cell entry. **(B)** Transient normalization window: improved perfusion and endothelial permissiveness promote T-cell trafficking into tumor nests. **(C)** Hypoxia/myeloid-rebound immunosuppressive state: excessive pruning induces hypoxic niches enriched with TAMs, MDSCs, and Tregs. **(D)** VEGF-independent vascular escape: vessel co-option, vasculogenic mimicry, and intussusceptive remodeling maintain perfusion and reduce sensitivity to VEGF-centered therapy.

An angio-immune ecosystem state is a reproducible, clinically meaningful configuration of the tumor microenvironment in which vascular function, immune contexture, and spatial tissue architecture are coupled and jointly constrain tumor evolution and therapy response (Kabir et al., 2025). Unlike conventional “angiogenesis signatures” (often centered on VEGF activity) or “immune phenotypes” (often centered on CD8^+^ abundance), a state is defined by interactions between axes—for example, how endothelial properties permit or block leukocyte arrest and transendothelial migration, how immune/myeloid programs reshape vessel behavior, and how spatial topology stabilizes immune exclusion or productive intratumoral immunity. Figure 1 provides an integrative spatial schematic of four representative angio-immune ecosystem states, linking distinct vascular phenotypes to endothelial gatekeeping, perivascular niche composition, stromal–parenchymal barriers, and immune-cell positioning within the tumor microenvironment. The figure further illustrates that vascular remodeling does not uniformly promote immune infiltration; instead, its immunological consequences depend on whether the local tissue architecture supports CD8^+^ T-cell trafficking into tumor nests, reinforces hypoxia/myeloid-driven suppression, or shifts toward VEGF-independent vascular escape (Kabir et al., 2025; Jain, 2005).

To make this framework spatially explicit, we conceptualize each angio-immune ecosystem state across five interconnected anatomical and functional zones. First, the intravascular lumen determines whether circulating lymphocytes can slow down, arrest, and become positioned for extravasation under abnormal tumor blood flow. Second, the endothelial layer functions as an active immune gatekeeper rather than a passive barrier: tumor endothelial cells can alter adhesion-molecule expression, chemokine presentation, antigen presentation, and inhibitory-ligand expression, thereby regulating leukocyte arrest and transendothelial migration. Third, the perivascular niche integrates endothelial cells, pericytes, macrophages, Tregs, fibroblasts, and soluble angiogenic or inflammatory cues, generating either permissive immune-entry sites or suppressive perivascular traps. Fourth, the stromal–parenchymal interface, shaped by CAFs and extracellular matrix remodeling, determines whether immune cells that have exited the vasculature can penetrate tumor nests or remain confined to stromal rims. Finally, the immune-cell trafficking axis connects these compartments into a dynamic process, from vascular adhesion to intratumoral positioning and effector function. Thus, immune exclusion should not be interpreted merely as low immune abundance, but as a spatial failure of trafficking, positioning, and functional activation.

Operationally, these spatial zones can be translated into three measurable dimensions that define each angio-immune ecosystem state. (i) Vascular performance/structure: perfusion and oxygenation (hypoxia), permeability, vessel density and caliber, endothelial activation versus “anergy,” and mural cell/pericyte coverage, features that jointly determine both drug delivery and the physical/molecular rules of immune trafficking (Amersfoort et al., 2022). (ii) Immune composition and polarization: the balance of cytotoxic T/NK activity versus suppressive populations (Tregs, MDSCs, immunosuppressive macrophages), and APC competence, which collectively dictates whether vascular changes translate into effective antitumor immunity (Subramanian et al., 2023). (iii) Spatial topology and immune positioning: where immune cells localize relative to vessels, stromal barriers, tumor nests, and TLS/HEV-like structures; whether CD8^+^ T cells penetrate malignant-cell neighborhoods or remain confined to stromal rims and perivascular compartments; and whether specialized niches, such as TLS-associated HEVs or perivascular suppressive zones, emerge to reinforce a given program (Palla et al., 2022; Vella et al., 2023).

Mechanistically, the “glue” holding these layers together is endothelial gatekeeping and reciprocal feedback. Classic work on tumor endothelial “anergy” showed that angiogenic signaling can suppress adhesion molecule induction and thereby reduce leukocyte–endothelium interactions; importantly, anti-angiogenic interventions can partially reverse this defect and increase immune infiltration, consistent with a normalization window that is real but often transient. Finally, the framework naturally accommodates escape transitions (VEGF-independent vascularization such as vessel co-option), where escalating VEGF blockade fails because the tumor is no longer VEGF-dependent (Frentzas et al., 2016). We do not intend this four-state framework to be exhaustive of all mechanisms of resistance to anti-angiogenic therapy or ICB. Rather, it focuses on recurrent vascular–immune ecosystem-level configurations that are measurable across bulk, single-cell, and spatial platforms. Tumor-intrinsic resistance mechanisms, including defects in antigen presentation, impaired interferon signaling, loss of tumor antigenicity, oncogenic pathway activation, epithelial–mesenchymal transition, metabolic rewiring, and therapy-induced clonal selection, may coexist with or drive transitions among these ecosystem states. Therefore, the proposed taxonomy should be viewed as a vascular–immune layer that complements, rather than replaces, tumor-intrinsic models of therapeutic resistance.

## 3 Angio-immune ecosystem states associated with therapeutic resistance: defining features and translational implications

### 3.1 Immune-excluded vascular–stromal barrier state: endothelial gatekeeping with spatial immune confinement

A clinically common failure mode for immunotherapy, especially in “T-cell present but ineffective” tumors, is an immune-excluded architecture in which CD8^+^ T cells accumulate in stromal rims or perivascular compartments yet remain physically separated from malignant cell neighborhoods. Rather than reflecting a single pathway defect, this state is best understood as endothelial gatekeeping coupled to stromal topology: abnormal vessels fail to provide permissive adhesion/chemokine cues for leukocyte arrest and diapedesis, while the remodeled extracellular matrix and activated fibroblast programs bias immune migration along stromal tracks rather than into tumor nests. Spatially, this state is centered on the endothelial interface and the stromal–parenchymal boundary, where impaired leukocyte adhesion, perivascular retention, and CAF/ECM-mediated confinement prevent CD8^+^ T cells from reaching malignant-cell neighborhoods. Mechanistically, VEGF signaling is a central node that links vascular dysfunction to immune exclusion: VEGF can suppress endothelial adhesion molecules, including ICAM and VCAM, alter chemokine presentation, and induce PD-L1/PD-L2 on endothelial cells, thereby weakening leukocyte arrest, transendothelial migration, T-cell trafficking, and effector function; it also impairs dendritic cell maturation and promotes immunosuppressive populations such as Tregs and MDSCs, creating a self-reinforcing exclusionary loop.

Multiple clinically practice-changing regimens can be interpreted as “attempts to break” this barrier state by simultaneously addressing immune activation and vascular permissiveness. In hepatocellular carcinoma (HCC), the IMbrave150 trial established atezolizumab plus bevacizumab as a first-line standard by improving survival compared with sorafenib, consistent with the idea that VEGF blockade can relieve vascular/immune suppression and improve immune access while PD-L1 blockade restores T-cell function (Ai et al., 2024; Tabuchi et al., 2025). In metastatic nonsquamous NSCLC, IMpower150 tested atezolizumab with bevacizumab and chemotherapy (carboplatin/paclitaxel), again leveraging anti-VEGF biology to “open” the microenvironment while checkpoint blockade amplifies cytotoxic immunity (Socinski et al., 2018). In renal cell carcinoma (RCC)—a classically VEGF-dependent disease—first-line combinations such as pembrolizumab–axitinib (KEYNOTE-426) and nivolumab–cabozantinib (CheckMate-9ER) provide concrete clinical proof that pairing ICI with VEGFR-targeting agents can overcome a barrier-like ecosystem in which vasculature and immunity are co-dysregulated (Wang Y. et al., 2022; Choueiri et al., 2021).

From a translational standpoint, the critical point is that this state is frequently misclassified if one only measures immune abundance. A tumor can score “inflamed” by bulk RNA or IHC counts yet behave as immune-excluded if immune cells are spatially trapped outside malignant neighborhoods. This is why spatial and multi-omics integration is not an add-on, but a requirement: this state is most faithfully operationalized using spatial transcriptomics or multiplex imaging to quantify immune–tumor distances, perivascular immune clustering, and stromal rim confinement, then anchored by endothelial activation programs (adhesion/chemokine modules) and functional vascular context (perfusion/hypoxia proxies). In practice, the clinically actionable implication is not simply “add an anti-VEGF,” but “titrate and schedule vascular modulation to convert spatial exclusion into intratumoral access,” because excessive vascular pruning can push tumors into a different resistance state. Similar inflamed-yet-excluded patterns are frequently encountered in high-grade serous ovarian cancer and recurrent cervical cancer, where CAF/ECM-rich stromal rims, perivascular trapping, and ascites-associated inflammation can leave bulk “immune-high” profiles clinically unresponsive. Therefore, state-informed integration of spatial metrics with vascular-function proxies may be particularly actionable in OB/GYN practice to guide scheduling/titration of vascular modulation before escalating to additional combination layers.

### 3.2 Transient normalization-window state: therapy-induced re-perfusion with time-limited immune permissiveness

A second, clinically underexploited ecosystem configuration is the transient “normalization window,” in which anti-angiogenic therapy partially restores vascular structure and function—improving perfusion and reducing hypoxia—thereby creating a time-limited interval during which immune infiltration and therapy delivery become more effective. Importantly, normalization is not the default outcome of VEGF pathway inhibition; it is dose-, tumor-, and time-dependent. The window can arise rapidly (even within ∼1 day in some settings) and is reversible; it can be short if treatment prunes vessels excessively, exacerbating hypoxia/acidosis and promoting immunosuppressive recruitment. In spatial terms, the normalization window represents a temporary reopening of the immune-cell trafficking axis: improved perfusion and endothelial permissiveness allow circulating lymphocytes to transition from the intravascular lumen, across the endothelial barrier, and into tumor-proximal or intratumoral compartments.

This state has unusually direct “instance-level” evidence linking vascular kinetics to therapeutic efficacy. Low-dose anti-VEGFR2 during a normalization interval can increase intratumoral T-cell infiltration, and that delivering immunotherapy (vaccination or adoptive transfer) during this window improves efficacy versus immunotherapy alone—explicitly demonstrating that timing and dose can change the immune geography of tumors (Bocca et al., 2017). Clinically, the principle that “vascular modulation can augment other modalities when correctly timed” is reflected not only in ICI combinations (IMbrave150, IMpower150, KEYNOTE-426, CheckMate-9ER) but also in broader oncology experience where vascular normalization improves delivery and oxygenation for cytotoxic therapy and radiotherapy.

Equally critical—and often neglected in review writing—is that the collapse of the normalization window is itself a resistance transition. The window closes when tumors adapt via alternative angiogenic pathways (e.g., ANG2–TIE2 signaling) or switch to alternative vascularization mechanisms such as vessel co-option. This is where concrete biomarker-linked instances become central: circulating ANG2 can rebound after VEGF pathway inhibition in glioblastoma, and elevated ANG2 has been associated with unfavorable responses to immune checkpoint blockade in melanoma; these observations motivate dual-axis strategies (VEGF + ANG2) to extend normalization and reprogram the immunosuppressive microenvironment (Schmittnaegel et al., 2017; Amoozgar et al., 2016).

The translational corollary is that “VEGF blockade” should not be treated as a binary on/off intervention but as a tunable control knob whose optimal setting may differ by baseline vascularity, hypoxia, and immune architecture—precisely the type of stratification problem that multi-omics and AI-assisted state modeling can formalize. Concrete drug-level examples show how the field is already moving toward non-VEGF single-axis thinking. Beyond classical bevacizumab, multiple VEGFR TKIs (axitinib, cabozantinib, lenvatinib, sorafenib) are used in combination with ICIs, and high-dose, prolonged VEGF pathway blockade can intensify hypoxia and drive CXCL12/SDF-1–CXCR4–mediated recruitment of immunosuppressive cells; in a mouse model of advanced HCC, high-dose sorafenib increased hypoxia and promoted a CXCR4-linked influx of Tregs and M2-like macrophages, while blocking CXCR4 mitigated that immunosuppressive shift. This mechanistic pattern is not merely theoretical: plerixafor (AMD3100), a CXCR4 inhibitor, has been evaluated in combination with bevacizumab in high-grade glioma as a clinically motivated attempt to counteract a CXCR4-driven resistance axis induced by VEGF pathway inhibition (Lee et al., 2018; Phase and Dana-Farber Cancer, 2011).

Clinical experience also shows that vascular–immune combinations are not universally effective. For example, atezolizumab added to fluoropyrimidine plus bevacizumab maintenance did not improve PFS or OS in BRAF-wild-type metastatic colorectal cancer in MODUL cohort 2, and bevacizumab-containing strategies in breast cancer, including AVADO and BEATRICE, showed limited or non-durable benefit (Tabernero et al., 2022; Cameron et al., 2013). These negative or modest results suggest that VEGF/VEGFR blockade can enhance ICB only when tumor-intrinsic antigenicity, endothelial state, myeloid/stromal context, and spatial immune positioning are permissive.

### 3.3 Myeloid-rebound immunosuppressive state: hypoxia-driven innate reprogramming and “adaptive immune relapse”

A third resistance-associated angio-immune ecosystem state emerges when initial vascular modulation and immune activation are followed by an innate immune rebound that restores angiogenesis and reinstates immunosuppression. This state is often precipitated by therapy-induced hypoxia and tissue stress, which act as ecological selection pressures that favor recruitment and reprogramming of myeloid populations (macrophages, monocytes, neutrophils) into proangiogenic and suppressive phenotypes. Continuous vessel pruning can generate hypoxic niches that recruit and polarize immunosuppressive and angiogenic myeloid cells, undermining durable benefit even when anti-VEGF therapy initially improves immune access. This rebound state is therefore spatially organized around hypoxic and perivascular niches, where suppressive macrophages, monocytes, neutrophils, and Tregs can accumulate near dysfunctional vessels and restore both pro-angiogenic signaling and T-cell-suppressive programs.

Here, specific experimental instances are instructive because they reveal a “respond → relapse” trajectory that maps cleanly onto a state-transition model. In the Rip1Tag2 pancreatic neuroendocrine tumor model, VEGF signaling blockade produced an early response characterized by reduced vessel density and apparent normalization with growth stasis, followed by relapse within weeks. Comparative profiling between responding versus relapsing tumors showed that innate immune cells shifted from angiostatic/immunostimulating features (associated with CD8 influx) back to immunosuppressive and angiogenic phenotypes in relapsing tumors, accompanied by cessation of CD8 infiltration. A key mechanistic switch implicated in this relapse was activation of the PI3Kγ pathway in myeloid cells, which disabled repolarization and promoted proangiogenic tumor relapse—an example that directly links a myeloid signaling node to a vascular–immune escape state.

This mechanistic axis has drug-development and trial-level relevance. The foundational Nature study on targeting PI3Kγ in myeloid cells demonstrated that inhibiting PI3Kγ can reshape the tumor immune microenvironment and overcome resistance to checkpoint blockade in preclinical settings, motivating clinical translation (De Henau et al., 2016). A concrete clinical instantiation of this concept is eganelisib (IPI-549), a PI3Kγ inhibitor evaluated in patients with advanced solid tumors in combination with PD-1/PD-L1 inhibitors, explicitly aiming to counteract myeloid-driven suppression that can dominate post-therapy relapse ecosystems (Hong et al., 2023; Texas MD Anderson Cancer Center, 2015). Parallel examples underscore that myeloid rebound is not limited to PI3Kγ. ANG2–TIE2 biology provides another “instance-rich” axis that couples endothelial signaling to myeloid behavior.

These mechanistic insights align with drug-level efforts to block both VEGF and ANG2: vanucizumab, a bispecific antibody targeting VEGF-A and ANG2, has been tested clinically in a first-in-human phase I study, illustrating how the field is operationalizing “dual-axis vascular control” to prevent escape through ANG2-driven remodeling (Hidalgo et al., 2018). At the level of translational implications, the myeloid-rebound state argues for a shift in how we define “resistance.” Instead of treating progression after ICI + anti-angiogenic therapy as a generic failure, this state posits a measurable ecological transition: increased hypoxia/stress signatures, accumulation of suppressive myeloid programs, reduced antigen presentation/effector function, and renewed angiogenic activity. This is precisely where multi-omics integration becomes necessary rather than decorative: bulk transcriptomics can flag hypoxia and myeloid modules, single-cell atlases can resolve specific suppressive macrophage/monocyte states, and spatial profiling can localize these populations to perivascular or stromal niches that mediate relapse. Clinically, the most actionable inference is that durable benefit may require triplet logic in selected patients—vascular modulation + ICI + myeloid reprogramming (e.g., PI3Kγ, CSF1R, CXCR4 axes)—deployed not upfront indiscriminately, but triggered by early biomarkers of rebound.

### 3.4 Alternative vascularization escape state (vessel co-option, vasculogenic mimicry, and intussusceptive remodeling)

A fourth, conceptually decisive resistance-associated ecosystem state arises when tumors escape VEGF-dependent sprouting angiogenesis and instead maintain perfusion through alternative vascularization programs that are intrinsically less sensitive to VEGF pathway blockade. Tumors can bypass classical sprouting via vessel co-option, vasculogenic mimicry, or intussusception, each sustaining blood supply under anti-angiogenic pressure. Vessel co-option is the most clinically substantiated of these mechanisms: because tumor cells hijack pre-existing host vessels rather than inducing VEGF-driven neovessels, co-option is inherently resistant to VEGF-targeted therapies. Unlike the normalization-window or endothelial-gatekeeping states, this escape state is primarily architectural: tumor perfusion is maintained through spatially distinct vascular patterns, including co-opted host vessels, tumor-cell-lined mimicry channels, or vascular splitting, making bulk angiogenesis scores insufficient for diagnosis. Histopathology remains the practical gold standard to distinguish co-option from angiogenesis, and co-opted liver metastases often display an immune-cold microenvironment with reduced immune infiltration compared with angiogenic lesions. Importantly, this is not merely conceptual: Frentzas et al. reported patient-level evidence in colorectal cancer liver metastases linking co-option to poor response to bevacizumab, directly connecting vascularization mode to clinical resistance (Frentzas et al., 2016).

Vasculogenic mimicry represents a distinct escape topology in which tumor cells form perfusable channel-like networks independent of endothelial cells, originally described in aggressive melanomas. These ECM-rich structures are associated with invasion, metastasis, poor prognosis, and resistance to anti-angiogenic therapy precisely because they are not VEGF-dependent. Although robust clinical biomarkers remain limited, this state is well suited to spatially resolved characterization: spatial transcriptomics can capture tumor-cell programs consistent with mimicry, and multiplex imaging can demonstrate CD31-negative, tumor-cell-lined channels *in situ*, supporting mechanistically grounded classification rather than inference from bulk “angiogenesis scores.” Intussusceptive angiogenesis (vascular splitting) provides a third route that can remodel vessels rapidly with minimal proliferation, and may act as a compensatory mechanism under anti-angiogenic pressure, helping explain preserved perfusion despite sustained VEGF inhibition. Because different vascular–immune states may require distinct therapeutic interventions, Table 1 summarizes major vascular-targeting and vascular–immune combination strategies according to their primary targets, expected vascular effects, immune consequences, relevant ecosystem states, and potential resistance clues.

**TABLE 1:** Resistance-associated angio-immune ecosystem states, key readouts, and state-matched therapeutic implications.

Therapeutic strategy	Representative agents	Primary target	Expected vascular effect	Immune consequence	Most relevant ecosystem state
VEGF-A neutralization	Bevacizumab; atezolizumab + bevacizumab	VEGF-A	Reduces VEGF-driven leakiness and endothelial suppression; may promote normalization within an optimal window	Improves T-cell trafficking and relieves VEGF-mediated immunosuppression	Immune-excluded barrier; normalization-window state
VEGFR2 blockade	Ramucirumab	VEGFR2	Blocks endothelial VEGF signaling and angiogenic activation	May reduce endothelial anergy and improve immune access, but immune effects are context-dependent	VEGF-dependent vascular barrier state
Selective VEGFR TKIs	Axitinib; sunitinib	VEGFR1/2/3 and related kinases	Inhibits angiogenic signaling; may normalize or prune vessels depending on dose/schedule	Can enhance ICI response but may also intensify hypoxia if excessive	Normalization-window state; hypoxia-rebound state
Multi-targeted TKIs	Lenvatinib; cabozantinib; sorafenib	VEGFR plus FGFR/MET/AXL/RAF or other kinases	Broader vascular and stromal pathway modulation	May affect myeloid recruitment, stromal remodeling, and T-cell infiltration beyond VEGF blockade	Mixed barrier/rebound states
Ang2/TIE2-axis targeting	Trebananib; vanucizumab; Ang2/VEGF dual blockade	ANG2/TIE2 ± VEGF-A	Stabilizes or normalizes vessels; may reduce abnormal remodeling	Can reduce pro-angiogenic myeloid support and improve immune permissiveness	Normalization-window collapse; myeloid-rebound state
ICI + anti-angiogenic combinations	Atezolizumab + bevacizumab; pembrolizumab + axitinib; nivolumab + cabozantinib	PD-1/PD-L1 plus VEGF/VEGFR pathways	Combines vascular modulation with checkpoint reinvigoration	Promotes T-cell entry and effector function when vascular state is permissive	Immune-excluded barrier; normalization-window state
Myeloid-reprogramming combinations	PI3Kγ inhibitors; CSF1R inhibitors; CXCR4 inhibitors with anti-VEGF/ICI	PI3Kγ, CSF1R, CXCR4	Indirectly modifies vascular rebound through innate immune remodeling	Reduces suppressive macrophage/monocyte recruitment and restores T-cell activity	Myeloid-rebound immunosuppressive state
Strategies for VEGF-independent escape	Anti-invasive or niche-remodeling approaches; pathology/spatially guided combinations	Co-option, mimicry, stromal/adhesion programs	Targets non-sprouting perfusion routes rather than VEGF-dependent angiogenesis	May require restoring immune recognition in co-opted or mimicry-rich regions	Alternative vascularization escape state

The translational implications are clear. First, this state explains why escalating VEGF blockade often fails: once perfusion becomes VEGF-independent, intensification may worsen hypoxia and foster immunosuppressive rebound without addressing the underlying supply strategy. Second, it shifts therapeutic logic toward pathways controlling invasion/adhesion and tissue hijacking—programs that plausibly enable co-option—and toward interventions that reshape local immune niches. Third, it underscores the need for ecosystem-state diagnostics, because co-option, mimicry, and intussusception are fundamentally spatial/architectural phenomena that require integrated pathology and spatial multi-omics for reliable detection and patient stratification.

## 4 Spatial and single-cell technologies reshaping mechanistic understanding of vascular–immune ecosystems

Spatially resolved and single-cell technologies have shifted vascular–immune biology from marker-based description to architecture-aware mechanism. Bulk RNA-seq can identify angiogenic, hypoxic, or inflammatory programs, and scRNA-seq can resolve cellular states, but both approaches lose the tissue coordinates that determine whether immune cells are intravascular, perivascularly trapped, stromally excluded, TLS-associated, or positioned within malignant-cell neighborhoods. Spatial transcriptomics platforms, including 10x Genomics Visium/Visium HD, Slide-seq, MERFISH, Xenium, CosMx, and STEREO-seq, bridge this gap by mapping gene-expression programs onto intact tumor architecture. Studies in hepatocellular carcinoma, glioma/glioblastoma, and colorectal cancer have shown that immune and stromal programs are regionally organized and that vessel-proximal or niche-specific neighborhoods can contain transcriptional states not captured by bulk profiling or dissociated single-cell analysis alone (Wang YF. et al., 2022; Ren et al., 2023; Greenwald et al., 2024; Oliveira et al., 2025). Thus, spatial transcriptomics supports the definition of angio-immune ecosystem states as local tissue configurations rather than isolated vascular or immune signatures.

Multiplex protein imaging provides a complementary layer because it measures cell phenotype and spatial proximity at single-cell resolution in intact tissue. Platforms such as CODEX/PhenoCycler, MIBI-TOF, imaging mass cytometry, cyclic immunofluorescence, and multiplex immunohistochemistry can quantify CD8^+^ T-cell distance to tumor nests, perivascular macrophage or Treg enrichment, endothelial-adjacent immune neighborhoods, and stromal exclusion patterns. These measurements convert broad concepts such as “immune infiltration,” “immune exclusion,” and “perivascular trapping” into measurable distances, neighborhoods, and cell–cell interaction patterns. For example, CODEX-based analysis in pancreatic ductal adenocarcinoma showed that CD8^+^ T cells may be present in lymphocyte-rich regions yet remain spatially distant from tumor cells, illustrating why immune abundance alone may misclassify an immune-excluded state (Xia et al., 2024). Similarly, integrated single-cell and spatial analyses in breast cancer identified FOLR2^+^ tissue-resident macrophages localized in perivascular stromal areas and associated with CD8^+^ T-cell infiltration, indicating that perivascular immune neighborhoods are functionally heterogeneous rather than uniformly suppressive (Nalio Ramos et al., 2022).

Single-cell endothelial atlases have further changed how tumor vasculature should be interpreted therapeutically. Earlier discussions of anti-angiogenic therapy often treated tumor endothelium as a relatively uniform target, whereas scRNA-seq studies now reveal diverse endothelial states, including angiogenic tip-like cells, stalk-like cells, venous and capillary endothelial cells, HEV-like endothelial cells, immunomodulatory endothelial states, and transitional or remodeling states. Lung cancer and pan-cancer endothelial atlases have shown conserved but context-dependent endothelial heterogeneity, including angiogenic CXCR4^+^ tip-like programs and inflammatory or venous-like programs with distinct immune associations (Goveia et al., 2020; Zhang et al., 2023). This heterogeneity is therapeutically important because VEGF/VEGFR blockade, Ang2/TIE2 targeting, or vascular-normalizing strategies may have different immune consequences depending on the dominant endothelial subset. The spatial context of tertiary lymphoid structures further reinforces this point. TLS are frequently associated with favorable prognosis and response to immune checkpoint blockade, but their formation and maintenance depend in part on specialized vascular structures, particularly high endothelial venules, which support lymphocyte recruitment and local immune organization (Vella et al., 2021). Therefore, tumor vessels should not be viewed only as abnormal conduits or barriers; depending on endothelial state and spatial context, they may either restrict immunity through endothelial anergy and stromal exclusion or support antitumor immunity through HEV/TLS-associated lymphocyte entry.

## 5 Building reproducible “state” stratifiers with multi-omics and lightweight AI

Given the field’s increasing emphasis on reproducible ecosystem states, the key to advancing the angio-immune ecosystem state concept lies in translating an initially descriptive framework into a transferable stratification tool, applicable across diverse cohorts, multiple technological platforms, and longitudinal time points. A practical strategy is to define each state using a small, testable signature stack—(i) vascular function modules, (ii) immune composition modules, and (iii) spatial/interaction readouts—then validate that the same state calls remain stable under cohort shifts and technical variation (Stuart et al., 2019; Korsunsky et al., 2019).

At the bulk level, state scoring can start with gene-set activity estimation rather than single markers, so that “perfusion–hypoxia tension,” endothelial activation/anergy, fibroblast barrier programs, and myeloid inflammation are each quantified as continuous axes rather than binary labels (Hänzelmann et al., 2013). Single-sample pathway scoring methods (e.g., GSVA/ssGSEA-like approaches) provide a simple, transparent way to compute these axes per patient and track transitions during therapy. To avoid confounding by changing cellularity, bulk signatures should be complemented by digital cytometry that estimates lineage proportions and, where possible, lineage-specific expression programs from bulk RNA-seq using single-cell references (Newman et al., 2019). This is especially valuable for angio-immune states where similar bulk “inflammation” can arise from distinct immune neighborhoods or myeloid phenotypes. Beyond cell fractions, ecosystem discovery frameworks can also infer co-occurring multicellular communities that behave as clinically meaningful “ecosystems,” offering a principled bridge from bulk cohorts to state labels (Luca et al., 2021).

To improve generalizability, single-cell atlases can be used to anchor the state definition to conserved endothelial and immune sub-states, enabling reference mapping of new cohorts and calibration of module weights (Stuart et al., 2019). Modern integration methods help align datasets across technologies and cohorts while preserving biological structure, which is essential if stratifier is expected to work across institutions (Korsunsky et al., 2019). Finally, building on the spatial mechanisms discussed above, a minimal ‘state’ tool should include spatial neighborhood quantification—distance-to-vessel/tumor, compartmental enrichment, and neighborhood graphs—implemented with scalable spatial-omics toolkits (Palla et al., 2022; Dries et al., 2021). When feasible, ligand–receptor inference can be added as a lightweight mechanistic layer to prioritize actionable communication edges (e.g., endothelial chemokine suppression, myeloid-stromal loops) that are consistent with the assigned state (Efremova et al., 2020).

## 6 Conclusions and outlook

Therapeutic resistance to vascular–immune combinations is frequently driven by ecosystem-level reconfiguration rather than isolated pathway escape. By framing resistance as transitions among a limited set of angio-immune ecosystem states—including vascular–stromal immune exclusion, transient normalization windows, myeloid rebound immunosuppression, and VEGF-independent alternative vascularization—this mini-review provides a unifying lens to reconcile heterogeneous clinical outcomes and to identify state-matched vulnerabilities. Importantly, these states are not abstract constructs: they are measurable using an integrated stack of bulk transcriptional modules, single-cell–anchored cell-state programs, and spatial neighborhood features that capture tissue architecture and immune trafficking constraints.

Looking ahead, the most impactful advances will come from converting state definitions into reproducible stratification tools supported by external validation, paired pre/post-treatment sampling, and transparent code and signature reporting. Clinically, this enables three practical goals: (i) timing immunotherapy to normalization-permissive intervals; (ii) early detection of myeloid rebound programs that warrant escalation to myeloid-targeting strategies; and (iii) recognition of VEGF-independent escape (e.g., vessel co-option or mimicry) that should trigger a shift away from intensified VEGF blockade toward anti-invasive and niche-remodeling approaches. Finally, because tumors often exhibit state mixtures across regions and metastatic sites, future studies should prioritize spatially resolved, longitudinal designs that quantify state dynamics at the lesion level. Collectively, a state-to-strategy framework can accelerate the translation of ecosystem modeling into actionable decision support for precision oncology.
